# Genetic Variation of Fatty Acid Oxidation and Obesity, A Literature Review

**Published:** 2016-03

**Authors:** Harry Freitag Luglio

**Affiliations:** Department of Nutrition and Health, Faculty of Medicine, Universitas Gadjah Mada, Indonesia

**Keywords:** obesity, genetic, fatty acid, lipolysis, metabolism

## Abstract

Modulation of fat metabolism is an important component of the etiology of obesity as well as individual response to weight loss program. The influence of lipolysis process had receives many attentions in recent decades. Compared to that, fatty acid oxidation which occurred after lipolysis seems to be less exposed. There are limited publications on how fatty acid oxidation influences predisposition to obesity, especially the importance of genetic variations of fatty acid oxidation proteins on development of obesity. The aim of this review is to provide recent knowledge on how polymorphism of genes related fatty acid oxidation is obtained. Studies in human as well as animal model showed that disturbance of genes related fatty acid oxidation process gave impact on body weight and risks to obesity. Several polymorphisms on CD36, CPT, ACS and FABP had been shown to be related to obesity either by regulating enzymatic activity or directly influence fatty acid oxidation process.

## INTRODUCTION

In this modern era, the awareness of obesity as important health risk is increasing. From several investigations on mechanism of obesity, systemic lipid metabolism appeared to be a hot topic. Studies done using different approaches rose a same agreement that disturbance in fat metabolism is not only a risk for developing of obesity but also an important factor that influence successfulness of weight loss program in obese individual. Previously, it was found that lipolysis in obese and overweight individual is impaired (1). Genetic studies showed that polymorphism of genes related lipolysis influenced weight loss in obese subjects during weight loss although results are still controversial (2-8). Furthermore, Rogge (9) proposed that fatty acid oxidation (FAO) is also an important factor that influences development of obesity in human. In the review, the author stated that the individual differences of FAO not only give contribution for development of obesity but also complicate weight loss treatment.

Recent studies demonstrated that the differences between healthy lean individual and obese in the term of energy metabolism included 4 factors: reduction of FAO, increasing demand of glucose for ATP synthesis, lower basal ATP concentration and accumulation of lipid in muscles and several other organs (9). Reduction of FAO was discovered to be related to nutritional status. Studies in human showed that pre-obese subjects and formerly obese subjects have lower fat oxidation than lean counterpart as shown by increasing respiratory quotients (RQ). Filozof *et al*. (10) compared 8 obese individuals who had lost BMI from more than 30 kg/m^2^ into 24.5 ± 1.0 kg/m^2^ with 8 never-obese individuals (24.4 ± 1.0 kg/m^2^). Under the same weight maintenance diet (50% carbohydrate, 30% fat and 20% protein) for 3 days, post-obese subjects had higher RQ showing less FAO compared to never-obese subjects with the comparable body weight.

RQ describe how much energy that is used from carbohydrate and this is important for weight maintenance. In a population-based study, Seidell *et al*. (11) showed that increasing RQ as well as reduction of FAO were related to weight gain. This lower FAO was found increasing dependency of glucose as the source of energy. One of the important factors that contribute to lower FAO in obese individuals has been investigated before. Simoneau *et al*. (12) studied changes in enzymatic activity of FAO at muscle tissue in obese and lean individuals. They showed that FAO related enzymes such as CPT (carnitine palmitoyl transferase) and CS (citrate synthase) (will be discussed later) were lower in obese subjects compared to lean subjects. Interestingly, they also reported that weight reduction by a very low calorie diet and behavioral changes did not affect the level of those enzymatic markers (12).

It has been suspected that changes in CPT activity had a big influence on the weight regulation due to its importance in regulating ATP production from fat. Ragge argued that reduction of CPT reduce uptake of fatty acid by mitochondria, promotes lipogenesis and therefore reduce energy supply for physical activity (9). This reduction of CPT then contributes to higher body weight and individual ability to regain weight after weight loss. This theory had a significant influence to support the findings that obese individuals are resistance to lose weight. As reported by many studies, reduction of body weight was followed by reduction of energy expenditure, thus gave the unwanted effect of given weight treatment. Study done by Elia *et al.* (13) proved that obese people had different response to starvation than their normal counterpart. In the starving condition obese subjects used less fatty acid and ketone bodies for ATP production than lean subjects while protein loss and oxidation in obese subjects were higher than normal subjects.

Ragge proposed an interesting theory on the interaction between obesity and low oxidation status. In the review, it was stated that sedentary characteristics of sedentary lifestyle from obese individual not only the cause of obesity. Instead, obese people tend to be more sedentary in order to achieve more favorable energy balance. As mitochondria have less ability to convert energy from fatty acid, and produce more lactate, obese individuals are more likely to experience fatigue. On the other hand, high AMP: ATP ratio as the result of lower oxidative capability also induce neuroendocrine signals thus make those people are continuously seeking energy-dense food (9).

## DISCUSSION

### The Fatty Acid Oxidation Pathway

Mitochondria are important place where fatty acid can be oxidized because it contains all enzymes that is necessary for FAO. FAO is started with transportation of fatty acid into mitochondria, followed by carnitine shuttle and ended with β-oxidation cycle. This fatty acid is taken from circulation after hydrolysis of triglyceride and lipoprotein by lipoprotein lipase (LPL) in endothelium (14). In cytosol, fatty acid should be transported into mitochondria and the length of carbon atom of each fatty acid defines how this molecule transported. Both long chain fatty acid (LCFA) and very long chain fatty acid (VLCFA) require transporters to enter mitochondria. Kiens (15) reviewed that at least three protein that involved in this transport system, those are: fatty acid transport protein (FATP), plasma membrane-bound fatty acid binding protein (FABPpm), and fatty acid translocase (FAT/CD36). FATP is a transmembrane protein which important not only to transport fatty acid but also to convert VLFA and LCFA into Acyl-CoA (14). Recent reports demonstrated that expression and activity of these LCFA/VLCFA transporter enzymes were changed due to certain conditions including diet, exercise and obesity. Long term high fat diet was successfully increase level FABPm protein in muscle tissue while carbohydrate rich diet reduce the level (16). Obese individuals had higher FABPm protein expression compared to lean subjects^12^ and this increment also found in male subjects in the exercise group (17).

In order to get into inner side of mitochondria, acyl-CoA that is produced by FATP should be converted into another form using carnitine shuttle system. This system is basically formation and deformation of acylcarnitine by the help of three important proteins CPT1 (carnitine palmytotransferase 1). CACT (Carnitine acylcarnitine translocase) and CPT2 (carnitine palmytotransferase 2). CPT1 converts Acyl-CoA into acylcarnitine at the outer membrane of mitochondria. Acylcarnitine thus transported through inner membrane of mitochondria by CACT and then converted again into acyl-coA by CPT2 (14). The importance of carnitine shift on entering fatty acid into mitochondria makes proteins in this system become important in the regulation of β-oxidation. Once Acyl-CoA inside mitochondria, oxidation initiated by acyl-coenzyme A dehydrogenase (ACAD) (14).

### Components of Fatty Acid Oxidation

**ACS.** Acyl-CoA synthetase (ACS) is an important enzyme that provides substrate for both lipogenesis and oxidative process. This metabolite is not functionally limited to fatty acid metabolism but also can alters many important process including insulin secretion and glucose transport (18, 19). In its protein structure, ACS has region for AMP binding site and region for fatty acid binding site (20). ACS has several isoforms and located differently. ACS1 is located in intrinsic membrane of endoplasmic reticulum (ER) and mitochondrial-associated membrane (MAM), ACS4 located in the MAM fraction and ACS5 is located in outer membrane of mitochondria. It was suggested that the different location of ACS isoform also influence its function in an independent pathway (18, 20). From those isoforms, ACS1 is suggested to be related to oxidation since its expression is increased by induction of Peroxisome Proliferator Activated Receptor α (PPAR-α) (21, 22).

**CD36.** CD36 is a lipid transport protein which involved in many cellular processes including FAO. This protein is expressed in several tissues such as adipose tissue, intestine and vascular endothelial (23). CD36 is composed by intracytoplasmic domains and extracellular domain. The hydrophobic region is located in extracellular domain which suggested has interaction with plasma membrane (24). Deficiency of CD36 had influence in fatty acid metabolism which is proven by lower rate of plasma fatty acid clearance after meal (25).

**FABP.** Fatty acid binding protein is an important protein which target fatty acid into FAO pathway. In general, this protein has high affinity to LCFA both saturated and unsaturated. FABP expressed almost in every tissues (26). Although the main function of FABP is diverse in different tissues, its function in each tissue varies accordingly. In adipose tissue FABP influences TG storage, inflammation as well as regulates fatty acid species in plasma (26). Expression of FABP in skeletal muscle is related to FAO and esterification of LCFA (27, 28). In the liver, FABP also brings its ligands to FAO pathway as well as interacts with transcription factor such as PPAR α (26, 29, 30).

**FATP.** Together with FABP and CD36, FATP helps fatty acids to find its way to oxidation pathway. Additionally, this protein also helps LCFA transferred into mitochondria by adding acyl-coA on the fatty acid carbon chain. This additional acyl-coA activates LCFA so CPT1 can use that as a substrate. This protein is membrane bound and expressed in skeletal muscle and adipose tissue (31-33). Located in mitochondria, the expression of FATP affects FAO. FATP1 is one of the six FATP isoform that is present in skeletal muscle which contains AMP-binding motif for transport function (34).

**CPT system.** Canitine Palmitoyltransferase (CPT) system is composed by two important proteins, CPT1 and CPT2. CPT1 located in outer membrane of mitochondria while CPT2 located inside mitochondrial. CPT1 processes acyl-CoA made by FATP into acylcarnitine by incorporating carnitine molecule. This acylcarnitine can enter the layer of mitochondria thus CPT2 convert that back into Acyl-CoA (35). Although located and functionally different in oxidation pathways, both proteins share similarity (CPT1-A and CPT2 share 50% of homology) (36). There are 3 isoforms of CPT1: CPT1-A (expressed in liver, kidney, lung and spleen); CPT1-B (expressed in skeletal muscle, heart, adipose tissue and testis); and CPT1-C (expressed in brain) (35).

### Control of Fatty Acid Oxidation

Schreurs *et al*. (37) previously described 3 ways in regulating β-oxidation through enzymes such as ACS and CPT1 as well as metabolite like malonyl-coA. Principally, FAO is regulated by early flow of CPT1 system (38, 39). Malonyl-CoA is a potent inhibitor of CPT1 which is catalized by ACC (Acyl-CoA Carboxylase) (38). The other regulator of FAO is hormones. There are an evidences showing that insulin and thyroid hormones were able to regulate sensitivity of CPT1A but not CPT1B to malonyl-CoA in the liver (40, 41). It seems that, differences in the regulation of CPT1A and CPT1B are tissue specific. For instance, in muscle tissue CPT1B is regulated by PPARα and retinoid X receptor.

ACC is an enzyme that involved in the regulation FAO in human body as well as regulation of lipogenic process. This enzyme is important because it produces melanyl-CoA, a potent CPT inhibitor. Thus activity of this enzyme is an important communication process between lipogenic signal and FAO signal (37). The regulation of ACC is done via AMPK and protein kinases that control this phosphorilation also control ACC activity (42). Furthermore, transcription factor that is related to cholesterol and carbohydrate metabolism are also known to regulate ACC. Sterol regulatory element binding protein (SERBP1c) was reported to modify ACC expression as the response to diet or hormonal signals (43-45). There is also a study showing that carbohydrate responsive element binding protein (ChREBP) is involved in regulation of ACC (46).

FAO can also be altered by environmental trigger such as diet and exercise. In the case of obesity, this becomes more important because it will define the successfulness of weight loss intervention. A review done by Kiens *et al*. (47) nicely discussed an environmental factors that influences FAO in human. Dietary pattern influence the availability of important nutrients in the body. The state of carbohydrate storage, is able to affect FAO in human body. Roepstorff *et al*. (48) reported that higher glycogen storage will lead to lower FAO. On the other hand, Helge *et al*. (49) investigated the effect of prolonged fat-rich diet on FAO. In the study, they found that fat rich diet increased fat-oxidative capacity after 7 weeks. There are some interesting points on how diet-exercise correlates with FAO. It has been reported that FAO is lower when an individual receive carbohydrate rich diet 3-5 days before exercise compared to those with fat-rich diet. Thus it was assumed that diet had important effect on the preference of energy source during exercise, rather than exercise itself (50-52).

Considering exercise is an important factor to control FAO, it is necessary to keep in mind that there is no guarantee increasing intensity will always accompanied by increasing of FAO. As demonstrated by Romijn *et al*. (53), total FA oxidation reach its highest point at 65% of maximal oxygen uptake. This data is taken based on comparison between 25%, 65% and 85% of maximal oxygen uptake during training. Several theories have been proposed to answer how exercise influences oxidation in human body. Firstly, the increment of capillary density is observed due to endurance training. There also report on how this training increased lipid binding proteins and some enzymes involved in FAO (15). The enzymes related lipolysis such as ATGL and HSL were also increased after training suggesting that capacity of skeletal muscle tissue to use stored fat as energy were increased (54).

The impact of exercise on FAO pathway and the regulation on oxidation process has already investigated before. In early 80’s, Holloszy and Coyle (55) reported that endurance training increased mitochondrial contents of skeletal muscle thus improve its ability to use fat as the source of energy. Following reports argued that quality of skeletal muscle also determines the ability of muscle tissue to better oxidize fatty acid. In an *in vitro* study, Sahlin *et al*. (56) showed that the proportion of type I fibres influenced FAO. The molecular control of FAO during exercise is still under investigation. However, several investigations have been made to clarify the role of proteins related oxidation on control of FAO. It was shown that expression of FAT/CD36 influenced FAO in mitochondria. An interesting report showing that mitochondrial density of CD36 is higher in slow-twitch muscle, type of muscle that had higher maximal capacity of FAO. This protein also increased about 63% after 120 min of aerobic exercise explaining the importance of this protein for balancing energy source during exercise (56-60).

### Genetic Polymorphism of Fatty Acid Oxidation Related Proteins and Weight Loss

Skeletal muscle is an important component of the body that regulates energy expenditure not only because of its ability to use glucose and fatty acids as the source of energy, but also because this tissue is 40-50% of total body mass in adults (61). There are several proteins that expressed in skeletal muscle cells and influence its ability to “burn” more fat into energy. Thus genetic variations of proteins in FAO pathway were reported to be related to several traits including obesity. Some investigations revealed that polymorphism of genes in this pathway were also related to ability of obese/overweight individuals to weight loss during lifestyle intervention.

**ACSL.** There are six ACSL (long chain acetyl-coA synthase) family members identified in human and their polymorphisms were reported to influence ACS ability to initiate FAO. Variation on rs2419621 has been shown to influence weight loss in obese individual (62). This finding was also accompanied by the fact that T allele in this region increased ACSL transcription level in muscle (62). Teng *et al*. (61) investigated the role this polymorphism on expression level of ACSL using electrophoretic mobility shift assay (EMSA). They demonstrated that T allele was able to form an E-box element upstream of the second ACSL5 isoform transcript start site while C allele of this variant lacked of the third E-box element. This formation of E-box element is related to increment in ACSL5 promoter activity, thus lack of this element reduced the activity (61).

Interestingly, several other genes have been reported to produce proteins with Acyl-coA synthetase activity including SAH, MACS1, MACS2 and MACS3 (63). Although it is not clear whether those genes are directly involved in the regulation of FAO in human skeletal muscle, the polymorphism of this genes were reported to influence on weight status. Genetic polymorphism of SAH influenced body mass index, lipid profile and waist hip ratio (63). The other studies found that the interaction between SAH and obesity is obesity related (65, 66). This finding was also confirmed by the other study showed that MACS2 polymorphism was also influence those phenotypes (64). Until today, there is not many data on the underlying mechanism of how this polymorphism is related to obesity or weight loss. And because this protein is involved in two contradicting processes, oxidation and lipogenesis, it is not simple to take a conclusion which parts that plays role the most in ACSL polymorphism.

**CD36.** Recently Love-Gregory and Abumrad (25) discussed the influence of CD36 gene polymorphism and obesity as well as obesity related complications. In the review, they stated that genetic variations of CD36 were not strongly associated with obesity. Polymorphism of CD36 gene was more likely to be associated with metabolic disorder such as abnormal FFA, HDL and LDL levels (67-69). One potential explanation of how the expression of this gene influenced metabolic profile had been investigated by Kennedy *et al* (70). Adipose tissue of CD36-/- mice was more insulin sensitive and had lower inflammation compared to wild type model shown lack of CD36 were metabolically protective.

There is a controversy on how genetic variations in CD36 influence obesity. A study done in European adolescent population showed that four SNPs in CD36 (rs3211867, rs3211883, rs3211908 and rs1527483) was related to obesity, BMI and percentage of body fat (71). This finding was inconsistent with study done by Choquet *et al*. (72) who found no significant correlation between those variations on obesity. This meta-analysis done from 3 European countries (German, French and Finland) showed no effect on this polymorphism on obesity. Interestingly, they also discovered that polymorphism of rs3211883 was associated with obesity but with opposite direction from that found by Bokor *et al* (71, 72).

An investigation done in Europe showed a promising result on how CD36 influence individual susceptibility to obesity. Corpeleijn *et al*. (73) provides an important finding that -178A>C polymorphism of CD36 had influence on fasting fat oxidation of 722 obese subjects from 7 countries including The Netherlands, Denmark, France, Spain, United Kingdom, Czech Republic, and Sweden. The relationship is still significant even after adjustment with fat mass, HOMA index and free fatty acid level. In contrast, a case control study on 30 obese and 30 non obese children tried to link between CD36 polymorphism and glucose metabolism. However this study showed no relationship between CD36 variants and glucose metabolism. This study also failed to show the effect of CD36 polymorphism and plasma level of CD36 (74). Perhaps this is due to the expression of this gene is that mainly on skeletal muscle or gastrointestinal tracks but not in circulation.

**FABP.** To elucidate the influence of liver FABP (L-FABP) on obesity and weight gain, recent investigation by Atshaves *et al*. (75) used L-FABP ablated mice model. In their study they observed that mice with loss of L-FABP gain more fat tissue mass as well as weight than the wild type. Additionally, mice with L-FABP ablation also reduced their fat oxidation. The authors argued that L-FABP KO mice with high-fat diet due to lack of fatty acid transport to liver, LCFA is allocated more on storage rather than oxidation. Another study using adipose FABP (A-FABP) and intestinal (I-FABP) also find the similar result that mice lack of this gene increased body weight compared to wild type mice (76, 77).

There is limited publication on how genetic variation in this gene influence body weight or obesity and several studies failed to prove this association. Hayakawa *et al*. (78) investigated the impact of alanine to threonine substitution at codon 54 of the fatty acid binding protein 2 (FABP2) gene on obesity and insulin resistance in 258 Japanese subjects. However, the result showed no correlation between this polymorphism and obesity as well as other metabolic abnormalities. In African American individuals, Lei *et al*. (79) also found no difference on the risk of obesity between subjects with FABP 2 Thr54 alleles but they had small increasing of BMI compared to subjects without Thr54 alleles.

Ala54Thr variant allele of FABP2 gene might influence metabolic rate of individual thus gives an impact on obesity risk. This hypothesis was tested by Takakura *et al.* (80), a group of scientists from Japan, who investigated the influence of genetic variations of FABP2 on Japanese obese women. In this study, 80 obese women participated in a lifestyle intervention including diet and exercise. Interestingly, subjects with Thr variant had lower resting metabolic rate and waist circumference after diet and physical exercise therapy and those without Thr variant. Subjects with Thr allele also reported higher body weight at age of 20 than subjects without Thr. Despite of lack of repetition and evidence support, examining the role of FABP in the response of weight loss therapy would be interesting in the future.

**CPT.** Robitaille *et a*l. (81) investigated the role of CPT1 gene on obesity though a diet-gene interaction. They sequenced CPT1 gene from French-Canadian population and found 14 genetic variation in CPT1A and 26 variations in CPT1B. From those variations CPT1B c.282-18C > T and p.E531K variants were related to obesity. Furthermore, CPT1A A275/A275 variant on high fat diet had higher BMI compared to those on low-fat diet. This confirmed that those variant is more affected by high-fat diet since subjects with T275 allele shown no influence of fat intake on obesity parameters.

A recent report done in Alaska Native Health Research showed the relationship between genetic variants of CPT1A and obesity traits. About 28 SNPs of CPT1A were analyzed from these 1141 Yup’ik Eskimo. From those SNPs, P479L SNP was shown to be related to several parameters of obesity including BMI, percentage of body fat, waist and tight circumference. Subjects with L479 allele had lower percentage of those obesity traits than those with P479 allele. Lemas *et al*. (82) also shown several SNPs that also involved in obesity phenotypes, such as thigh circumference (rs2278908, rs2278907, P479L, rs4930248, rs11228372, rs11228373, and rs3019594), hip circumference (rs2278907, rs11228372, rs11228373 and rs3019594). This study confirmed previous clinical investigation of L479 allele of P479L variant in CPT1A gene done by Brown *et al*. (83). In an observation of six L-CPTI deficient patients they demonstrated that L479 allele diminished CPT1A enzyme activity in fibroblast cells as well as the ability of malonyl-coA to inhibit CPT1A.

## CONCLUSION AND FUTURE REMARKS

It was evident that fatty acid oxidation is an important process that influence the ability of body to reduce fat by diet and physical activity. Changes in this process were also linked to occurrence of obesity and several metabolic diseases. Studies in human as well as animal model showed that disturbance of genes related fatty acid oxidation process gave impact on body weight and risks to obesity. Several polymorphisms on CD36, CPT, ACS and FABP had been shown to be related to obesity either by regulating enzymatic activity or directly influence fatty acid oxidation process. However, those studies are lack of repetition and in some cases the findings were controversial. Further studies are necessary to clarify potent SNPs on those genes and the interaction with environmental factors including diet and physical activity, two processes that is tightly involved in fatty acid oxidation process. It will also be interesting to study the effect of high fat isocaloric diet on individual with certain genetic variations on FAO pathway to cross validate the diet-gene interaction in weight loss program.

## References

[1] Schiffelers SL, Saris WH, Boomsma F, van Baak MA (2001). beta(1)- and beta(2)-Adrenoceptor-mediated thermogenesis and lipid utilization in obese and lean men. J. Clin. Endocrinol. Metab.

[2] Corella D, Qi L, Sorli JV, Godoy D (2005). Obese subjects carrying the 11482G>A polymorphism at the perilipin locus are resistant to weight loss after dietary energy restriction. J. Clin. Endocrinol. Metab.

[3] Jang YKO, Lee JH, Koh SJ, Chae JS (2006). Genetic variation at the perilipin locus is associated with changes in serum free fatty acids and abdominal fat following mild weight loss. International journal of obesity.

[4] Deram S, Nicolau CY, Perez-Martinez P, Guazzelli I (2008). Effects of perilipin (PLIN) gene variation on metabolic syndrome risk and weight loss in obese children and adolescents. J. Clin. Endocrinol Metab.

[5] Soenen S, Mariman EC, Vogels N, Bouwman FG (2009). Relationship between perilipin gene polymorphisms and body weight and body composition during weight loss and weight maintenance. Physiol. Behav.

[6] Tchernof A, Starling RD, Turner A, Shuldiner AR (2000). Impaired capacity to lose visceral adipose tissue during weight reduction in obese postmenopausal women with the Trp64Arg beta3-adrenoceptor gene variant. Diabetes.

[7] Kim OY, Cho EY, Park HY, Jang Y (2004). Additive effect of the mutations in the beta3-adrenoceptor gene and UCP3 gene promoter on body fat distribution and glycemic control after weight reduction in overweight subjects with CAD or metabolic syndrome. Int. J. Obes. Relat. Metab. Disord.

[8] Lee JS, Kawakubo K, Inoue S, Akabayashi A (2006). Effect of beta(3)-adrenergic receptor gene polymorphism on body weight change in middle-aged, overweight women. Environ. Health Prev. Med.

[9] Rogge MM (2009). The role of impaired mitochondrial lipid oxidation in obesity. Biol. Res. Nurs.

[10] Filozof CM, Murua C, Sanchez MP, Brailovsky C (2000). Low plasma leptin concentration and low rates of fat oxidation in weight-stable post-obese subjects. Obes. Res.

[11] Seidell JC, Muller DC, Sorkin JD, Andres R (1992). Fasting respiratory exchange ratio and resting metabolic rate as predictors of weight gain: the Baltimore Longitudinal Study on Aging. Int. J. Obes. Relat. Metab. Disord.

[12] Simoneau JA, Veerkamp JH, Turcotte LP, Kelley DE (1999). Markers of capacity to utilize fatty acids in human skeletal muscle: relation to insulin resistance and obesity and effects of weight loss. FASEB J.

[13] Elia M, Stubbs RJ, Henry CJ (1999). Differences in fat, carbohydrate, and protein metabolism between lean and obese subjects undergoing total starvation. Obes. Res.

[14] Houten SM, Wanders RJ (2010). A general introduction to the biochemistry of mitochondrial fatty acid beta-oxidation. J. Inherit. Metab. Dis.

[15] Kiens B (2006). Skeletal muscle lipid metabolism in exercise and insulin resistance. Physiol. Rev.

[16] Roepstorff C, Helge JW, Vistisen B, Kiens B (2004). Studies of plasma membrane fatty acid-binding protein and other lipid-binding proteins in human skeletal muscle. Proc. Nutr. Soc.

[17] Kiens B, Kristiansen S, Jensen P, Richter EA, Turcotte LP (1997). Membrane associated fatty acid binding protein (FABPpm) in human skeletal muscle is increased by endurance training. Biochem. Biophys. Res. Commun.

[18] Coleman RA, Lewin TM, Van Horn CG, Gonzalez-Baro MR (2002). Do long-chain acyl-CoA synthetases regulate fatty acid entry into synthetic versus degradative pathways?. J. Nutr.

[19] Faergeman NJ, Knudsen J (1997). Role of long-chain fatty acyl-CoA esters in the regulation of metabolism and in cell signalling. Biochem J.

[20] Coleman RA, Lewin TM, Muoio DM (2000). Physiological and nutritional regulation of enzymes of triacylglycerol synthesis. Annu. Rev. Nutr.

[21] Schoonjans K, Watanabe M, Suzuki H, Mahfoudi A (1995). Induction of the acyl-coenzyme A synthetase gene by fibrates and fatty acids is mediated by a peroxisome proliferator response element in the C promoter. J. Biol. Chem.

[22] Schoonjans K, Staels B, Grimaldi P, Auwerx J (1993). Acyl-CoA synthetase mRNA expression is controlled by fibric-acid derivatives, feeding and liver proliferation. Eur. J. Biochem.

[23] Silverstein RL, Febbraio M (2009). CD36, a scavenger receptor involved in immunity, metabolism, angiogenesis, and behavior. Sci Signal 2, re3.

[24] Rac ME, Safranow K, Poncyljusz W (2007). Molecular basis of human CD36 gene mutations. Mol. Med.

[25] Love-Gregory L, Abumrad NA (2011). CD36 genetics and the metabolic complications of obesity. Curr Opin. Clin. Nutr. Metab. Care.

[26] Storch J, Thumser AE (2010). Tissue-specific functions in the fatty acid-binding protein family. J. Biol. Chem.

[27] Binas B, Erol E (2007). FABPs as determinants of myocellular and hepatic fuel metabolism. Mol. Cell. Biochem.

[28] Erol E, Cline GW, Kim JK, Taegtmeyer H (2004). Nonacute effects of H-FABP deficiency on skeletal muscle glucose uptake *in vitro*. Am. J. Physiol. Endocrinol. Metab.

[29] Hostetler HA, McIntosh AL, Atshaves BP, Storey SM (2009). L-FABP directly interacts with PPARalpha in cultured primary hepatocytes. J. Lipid. Res.

[30] Wolfrum C (2007). Cytoplasmic fatty acid binding protein sensing fatty acids for peroxisome proliferator activated receptor activation. Cell Mol. Life Sci.

[31] Sebastian D, Guitart M, Garcia-Martinez C, Mauvezin C (2009). Novel role of FATP1 in mitochondrial fatty acid oxidation in skeletal muscle cells. J. Lipid. Res.

[32] Hall AM, Smith AJ, Bernlohr DA (2003). Characterization of the Acyl-CoA synthetase activity of purified murine fatty acid transport protein 1. J. Biol. Chem.

[33] Bonen A, Miskovic D, Kiens B (1999). Fatty acid transporters (FABPpm, FAT, FATP) in human muscle. Can. J. Appl. Physiol.

[34] Stuhlsatz-Krouper SM, Bennett NE, Schaffer JE (1998). Substitution of alanine for serine 250 in the murine fatty acid transport protein inhibits long chain fatty acid transport. J. Biol. Chem.

[35] Bonnefont JP, Djouadi F, Prip-Buus C, Gobin S (2004). Carnitine palmitoyltransferases 1 and 2: biochemical, molecular and medical aspects. Mol. Aspects Med.

[36] Kolodziej MP, Zammit VA (1993). Mature carnitine palmitoyltransferase I retains the N-terminus of the nascent protein in rat liver. FEBS Lett.

[37] Schreurs M, Kuipers F, van der Leij FR (2010). Regulatory enzymes of mitochondrial beta-oxidation as targets for treatment of the metabolic syndrome. Obes. Rev.

[38] McGarry JD, Brown NF (1997). The mitochondrial carnitine palmitoyltransferase system. From concept to molecular analysis. Eur. J. Biochem.

[39] Eaton S (2002). Control of mitochondrial beta-oxidation flux. Prog. Lipid Res.

[40] Park EA, Mynatt RL, Cook GA, Kashfi K (1995). Insulin regulates enzyme activity, malonyl-CoA sensitivity and mRNA abundance of hepatic carnitine palmitoyltransferase-I. Biochem. J.

[41] Saggerson ED, Carpenter CA (1981). Effects of fasting and malonyl CoA on the kinetics of carnitine palmitoyltransferase and carnitine octanoyltransferase in intact rat liver mitochondria. FEBS Lett.

[42] Winder WW, Hardie DG (1996). Inactivation of acetyl-CoA carboxylase and activation of AMP-activated protein kinase in muscle during exercise. Am. J. Physiol.

[43] Magana MM, Lin SS, Dooley KA, Osborne TF (1997). Sterol regulation of acetyl coenzyme A carboxylase promoter requires two interdependent binding sites for sterol regulatory element binding proteins. J. Lipid Res.

[44] Shimomura I, Bashmakov Y, Ikemoto S, Horton JD (1999). Insulin selectively increases SREBP-1c mRNA in the livers of rats with streptozotocin-induced diabetes. Proc. Natl. Acad. Sci. USA.

[45] Oh SY, Park SK, Kim JW, Ahn YH (2003). Acetyl-CoA carboxylase beta gene is regulated by sterol regulatory element-binding protein-1 in liver. J. Biol. Chem.

[46] Ishii S, Iizuka K, Miller BC, Uyeda K (2004). Carbohydrate response element binding protein directly promotes lipogenic enzyme gene transcription. Proc. Natl. Acad. Sci. USA.

[47] Kiens B, Alsted TJ, Jeppesen J (2011). Factors regulating fat oxidation in human skeletal muscle. Obesity reviews : an official journal of the International Association for the Study of Obesity.

[48] Roepstorff C, Halberg N, Hillig T, Saha AK (2005). Malonyl-CoA and carnitine in regulation of fat oxidation in human skeletal muscle during exercise. Am. J. Physiol. Endocrinol. Metab.

[49] Helge JW, Kiens B (1997). Muscle enzyme activity in humans: role of substrate availability and training. Am. J. Physiol.

[50] EH C (1938). Albeitsfahigkeit und Ernahrung. Skandinavisches Archiv. Für. Physiologie.

[51] Helge JW, Richter EA, Kiens B (1996). Interaction of training and diet on metabolism and endurance during exercise in man. J. Physiol.

[52] Galbo H, Holst JJ, Christensen NJ (1979). The effect of different diets and of insulin on the hormonal response to prolonged exercise. Acta. Physiol. Scand.

[53] Romijn JA, Coyle EF, Sidossis LS, Gastaldelli A (1993). Regulation of endogenous fat and carbohydrate metabolism in relation to exercise intensity and duration. Am. J. Physiol.

[54] Alsted TJ, Nybo L, Schweiger M, Fledelius C (2009). Adipose triglyceride lipase in human skeletal muscle is upregulated by exercise training. Am. J. Physiol. Endocrinol. Metab.

[55] Holloszy JO, Coyle EF (1984). Adaptations of skeletal muscle to endurance exercise and their metabolic consequences. J. Appl. Physiol.

[56] Sahlin K, Harris RC (2008). Control of lipid oxidation during exercise: role of energy state and mitochondrial factors. Acta. Physiol. (Oxf).

[57] Sahlin K, Mogensen M, Bagger M, Fernstrom M (2007). The potential for mitochondrial fat oxidation in human skeletal muscle influences whole body fat oxidation during low-intensity exercise. Am. J. Physiol. Endocrinol. Metab.

[58] Holloway GP, Bezaire V, Heigenhauser GJ, Tandon NN (2006). Mitochondrial long chain fatty acid oxidation, fatty acid translocase/CD36 content and carnitine palmitoyltransferase I activity in human skeletal muscle during aerobic exercise. J. Physiol.

[59] Campbell SE, Tandon NN, Woldegiorgis G, Luiken JJ (2004). A novel function for fatty acid translocase (FAT)/CD36: involvement in long chain fatty acid transfer into the mitochondria. J. Biol. Chem.

[60] Mogensen M, Sahlin K (2005). Mitochondrial efficiency in rat skeletal muscle: influence of respiration rate, substrate and muscle type. Acta. Physiol. Scand.

[61] Teng AC, Adamo K, Tesson F, Stewart AF (2009). Functional characterization of a promoter polymorphism that drives ACSL5 gene expression in skeletal muscle and associates with diet-induced weight loss. FASEB journal: official publication of the Federation of American Societies for Experimental Biology.

[62] Adamo KB, Dent R, Langefeld CD, Cox M (2007). Peroxisome proliferator-activated receptor gamma 2 and acyl-CoA synthetase 5 polymorphisms influence diet response. Obesity.

[63] Iwai N, Katsuya T, Mannami T, Higaki J (2002). Association between SAH, an acyl-CoA synthetase gene, and hypertriglyceridemia, obesity, and hypertension. Circulation.

[64] Iwai N, Mannami T, Tomoike H, Ono K (2003). An acyl-CoA synthetase gene family in chromosome 16p12 may contribute to multiple risk factors. Hypertension.

[65] Benjafield AV, Iwai N, Ishikawa K, Wang WY (2003). Overweight, but not hypertension, is associated with SAH polymorphisms in Caucasians with essential hypertension. Hypertension research : official journal of the Japanese Society of Hypertension.

[66] Telgmann R, Brand E, Nicaud V, Hagedorn C (2007). SAH gene variants are associated with obesity-related hypertension in Caucasians: the PEGASE Study. Journal of hypertension.

[67] Ma X, Bacci S, Mlynarski W, Gottardo L (2004). A common haplotype at the CD36 locus is associated with high free fatty acid levels and increased cardiovascular risk in Caucasians. Human molecular genetics.

[68] Goyenechea E, Collins LJ, Parra D, Liu G (2008). CD36 gene promoter polymorphisms are associated with low density lipoprotein-cholesterol in normal twins and after a low-calorie diet in obese subjects. Twin research and human genetics: the official journal of the International Society for Twin Studies.

[69] Morii T, Ohno Y, Kato N, Hirose H (2009). CD36 single nucleotide polymorphism is associated with variation in low-density lipoprotein-cholesterol in young Japanese men. Biomarkers: biochemical indicators of exposure, response, and susceptibility to chemicals.

[70] Kennedy DJ, Kuchibhotla S, Westfall KM, Silverstein RL (2011). A CD36-dependent pathway enhances macrophage and adipose tissue inflammation and impairs insulin signalling. Cardiovascular research.

[71] Bokor S, Legry V, Meirhaeghe A, Ruiz JR (2010). Single-nucleotide polymorphism of CD36 locus and obesity in European adolescents. Obesity.

[72] Choquet H, Labrune Y, De Graeve F, Hinney A (2011). Lack of association of CD36 SNPs with early onset obesity: a meta-analysis in 9,973 European subjects. Obesity.

[73] Corpeleijn E, Petersen L, Holst C, Saris WH (2010). Obesity-related polymorphisms and their associations with the ability to regulate fat oxidation in obese Europeans: the NUGENOB study. Obesity.

[74] Rac ME, Krupa B, Garanty-Bogacka B, Syrenicz M (2012). Polymorphism of CD36 gene, carbohydrate metabolism and plasma CD36 concentration in obese children. A preliminary study. Postepy higieny i medycyny doswiadczalnej.

[75] Atshaves BP, McIntosh AL, Storey SM, Landrock KK (210). High dietary fat exacerbates weight gain and obesity in female liver fatty acid binding protein gene-ablated mice. Lipids.

[76] Hotamisligil GS, Johnson RS, Distel RJ, Ellis R (1996). Uncoupling of obesity from insulin resistance through a targeted mutation in aP2, the adipocyte fatty acid binding protein. Science.

[77] Vassileva G, Huwyler L, Poirier K, Agellon LB (2000). The intestinal fatty acid binding protein is not essential for dietary fat absorption in mice. FASEB journal: official publication of the Federation of American Societies for Experimental Biology.

[78] Hayakawa T, Nagai Y, Nohara E, Yamashita H (1999). Variation of the fatty acid binding protein 2 gene is not associated with obesity and insulin resistance in Japanese subjects. Metabolism: clinical and experimental.

[79] Lei HH, Coresh J, Shuldiner AR, Boerwinkle E (1999). Variants of the insulin receptor substrate-1 and fatty acid binding protein 2 genes and the risk of type 2 diabetes, obesity, and hyperinsulinemia in African-Americans: the Atherosclerosis Risk in Communities Study. Diabetes.

[80] Takakura Y, Yoshioka K, Umekawa T, Kogure A (2005). Thr54 allele of the FABP2 gene affects resting metabolic rate and visceral obesity. Diabetes research and clinical practice.

[81] Robitaille J, Houde A, Lemieux S, Perusse L (2007). Variants within the muscle and liver isoforms of the carnitine palmitoyltransferase I (CPT1) gene interact with fat intake to modulate indices of obesity in French-Canadians. Journal of molecular medicine.

[82] Lemas DJ, Wiener HW, O’Brien DM, Hopkins S (2012). Genetic polymorphisms in carnitine palmitoyltransferase 1A gene are associated with variation in body composition and fasting lipid traits in Yup’ik Eskimos. Journal of lipid research.

[83] Brown NF, Mullur RS, Subramanian I, Esser V (2001). Molecular characterization of L-CPT I deficiency in six patients: insights into function of the native enzyme. Journal of lipid research.

